# Supplementary material for: Oral Charcoal Adsorbents Attenuate Neointima Formation of Arteriovenous Fistulas
Source: Toxins (Basel). 2020 Apr 8;12(4):237. doi: 10.3390/toxins12040237 (PMC7232464; doi:10.3390/toxins12040237)
Supplement: Supplementary file 1 [file toxins-12-00237-s001.pdf]

Supplementary Material: Oral Charcoal Adsorbents Attenuate Neointima Formation of Arteriovenous Fistulas

Yu-Chung Shih, Chih-Cheng Wu, Shen-Chih Wang, Jun-Yang Liou, Po-Hsun Huang and Der-Cherng Tarng

Table S1. Primers for Quantitative Real-time Polymerase Chain Reaction.

Gene	Direction	Primer Sequence (5'-3')
TNF-α	Foward	GCCTCTTCTCATTCCTGCTTG
	Reverse	CTGATGAGAGGGAGGCCATT
MMP-2	Foward	AGATCTTCTTCTTCAAGGACCGGTT
	Reverse	GGCTGGTCAGTGGCTTGGGGTA
MMP-9	Foward	GTTTTTGATGCTATTGCTGAGATCCA
	Reverse	CCCACATTTGACGTCCAGAGAAGAA
MCP-1	Foward	GGCTCAGCCAGATGCAGTTAA
	Reverse	CCTACTCATTGGGATCATCTTGCT
IL-6	Foward	ACGGCCTTCCCTACTTCACA
	Reverse	CATTTCCACGATTTCCCAGA
HIF-1α	Foward	ACCTTCATCGGAAACTCCAAAG
	Reverse	CTGTTAGGCTGGGAAAAGTTAGG
VEGF-A	Foward	ATGAAGTGATCAAGTTCATGG
	Reverse	GGATCTTGGACAAACAAATGC
TGF-β1	Foward	GGACTCTCCACCTGCAAGAC
	Reverse	GACTGGCGAGCCTTAGTTTG
